# Supplementary material for: The Small RNA ErsA Plays a Role in the Regulatory Network of Pseudomonas aeruginosa Pathogenicity in Airway Infections
Source: mSphere. 2020 Oct 14;5(5):e00909-20. doi: 10.1128/mSphere.00909-20 (PMC7565897; doi:10.1128/mSphere.00909-20)
Supplement: TABLE S1 [file mSphere.00909-20-st001.pdf]

S1 Table. Analysis of ErsA expression in a panel of clinical and environmental strains of *P. aeruginosa*.

Strain	Origin [a]	Reference	Lane [b]	*ersA gene* detection by PCR	Differential *ersA* expression relative to PAO1	% [c]
MI1-5	CF	(1)	5	+	upregulation	8.3
MI3-2	CF	(1)	15	+		
TR67	CF	(2)	38	+		
MI1-1	CF	(1)	1	+	not significantly different	55.6
MI1-2	CF	(1)	2	+		
MI2-2	CF	(1)	8	+		
MI2-4	CF	(1)	10	+		
MI2-5	CF	(1)	11	+		
MI3-1	CF	(1)	14	+		
MI4	CF	(1)	16	+		
MI5	COPD	(1)	17	+		
MI6-1	CF	(1)	18	+		
MI7	CF	(1)	20	+		
MI10	COPD	(1)	23	+		
BT2	CF	(2)	25	+		
RP73	CF	(3)	28	+		
AA43	CF	(2)	33	+		
AA44	CF	(2)	34	+		
E1	E	(2)	39	+		
E2	E	(2)	40	+		
E4	E	(2)	41	+		
E5	E	(2)	42	+		
E9	E	(2)	43	+		
MI1-3	CF	(1)	3	-	downregulation or no expression	36.1
MI1-4	CF	(1)	4	+		
MI1-6	CF	(1)	6	+		
MI2-1	CF	(1)	7	+		
MI2-3	CF	(1)	9	+		
MI6-2	CF	(1)	19	+		
MI8	CF	(1)	21	+		
MI9	CF	(1)	22	+		
KK1	CF	(2)	24	+		
AA2	CF	(2)	32	+		
LESB58	CF	(2)	35	+		
TR1	CF	(2)	36	+		
TR66	CF	(2)	37	+		

[a] CF: Cystic Fibrosis patient; COPD: Chronic Obstructive Pulmonary Disease patient; E: Environment.
[b] Lane number in Figure 5.
[c] Percentage of strains with the indicated ersA expression.

References

1. Ferrara S, Falcone M, Macchi R, Bragonzi A, Girelli D, Cariani L, et al. The PAPI-1 pathogenicity island-encoded small RNA PesA influences Pseudomonas aeruginosa virulence and modulates pyocin S3 production. PLoS ONE. 2017;12(6).

2. Bragonzi A, Paroni M, Nonis A, Cramer N, Montanari S, Rejman J, et al. Pseudomonas aeruginosa microevolution during cystic fibrosis lung infection establishes clones with adapted virulence. Am J Respir Crit Care Med. 2009;180(2):138-45.

3. Bianconi I, Jeukens J, Freschi L, Alcala-Franco B, Facchini M, Boyle B, et al. Comparative genomics and biological characterization of sequential Pseudomonas aeruginosa isolates from persistent airways infection. BMC Genomics. 2015;16:1105.
